# Toward a Semi-Self-Paced EEG Brain Computer Interface: Decoding Initiation State from Non-Initiation State in Dedicated Time Slots

**DOI:** 10.1371/journal.pone.0088915

**Published:** 2014-02-21

**Authors:** Lingling Yang, Howard Leung, David A. Peterson, Terrence J. Sejnowski, Howard Poizner

**Affiliations:** 1 Department of Computer Science, City University of Hong Kong, Hong Kong; 2 Institute for Neural Computation, University of California San Diego, La Jolla, California, United States of America; 3 Computational Neurobiology Laboratory, Howard Hughes Medical Institute, The Salk Institute for Biological Studies, La Jolla, California, United States of America; 4 Division of Biological Sciences, University of California San Diego, La Jolla, California, United States of America; 5 Graduate Program in Neurosciences, University of California San Diego, La Jolla, California, United States of America; University of Adelaide, Australia

## Abstract

Brain computer interfaces (BCIs) offer a broad class of neurologically impaired individuals an alternative means to interact with the environment. Many BCIs are “synchronous” systems, in which the system sets the timing of the interaction and tries to infer what control command the subject is issuing at each prompting. In contrast, in “asynchronous” BCIs subjects pace the interaction and the system must determine when the subject’s control command occurs. In this paper we propose a new idea for BCI which draws upon the strengths of both approaches. The subjects are externally paced and the BCI is able to determine when control commands are issued by decoding the subject’s intention for initiating control in dedicated time slots. A single task with randomly interleaved trials was designed to test whether it can be used as stimulus for inducing initiation and non-initiation states when the sensory and motor requirements for the two types of trials are very nearly identical. Further, the essential problem on the discrimination between initiation state and non-initiation state was studied. We tested the ability of EEG spectral power to distinguish between these two states. Among the four standard EEG frequency bands, beta band power recorded over parietal-occipital cortices provided the best performance, achieving an average accuracy of 86% for the correct classification of initiation and non-initiation states. Moreover, delta band power recorded over parietal and motor areas yielded a good performance and thus could also be used as an alternative feature to discriminate these two mental states. The results demonstrate the viability of our proposed idea for a BCI design based on conventional EEG features. Our proposal offers the potential to mitigate the signal detection challenges of fully asynchronous BCIs, while providing greater flexibility to the subject than traditional synchronous BCIs.

## Introduction

Brain computer interfaces (BCIs), more generally known as brain-machine interfaces (BMIs), offer an alternative means to interact with the environment, which is particularly important for patients whose normal means of interaction are compromised by central nervous system damage, such as caused by brainstem strokes, upper spinal cord injury, and amyotrophic lateral sclerosis (ALS) [1]–[4]. They may also provide an assistive mechanism to healthy individuals in particularly demanding tasks [5]–[7]. Most laboratory-based BCIs are designed to determine what the subject’s intentions are, in the form of commands (or communication) signals, in a protocol where subjects are constantly prompted to make choices. These designs side-step the critical question of whether the subject is making a choice. The resulting BCI provides a communication protocol that can be unnatural, mentally demanding, and a source of user frustration [8]. In response, several groups have developed “asynchronous” BCIs [9], [10], many of which are completely self-paced [11]–[14]. In these cases, the subject volitionally determines when to issue commands, and the BCI must determine on an ongoing basis whether the subject is in a state of “intentional control” or “no control” (sometimes referred to as the “zero class”) [15]. In the extreme case, subjects have control over the onset and duration of the commands, in addition to specifying the commands themselves [16]. Asynchronous BCIs offer the advantage of increased usability because the subject has more control over the speed of communications. However, the BCI must then solve the signal detection problem, trying to maximize true positives while minimizing false positives, a tradeoff usually optimized by adopting extra layers of design complexity such as error correction schemes [17] and/or dwell times and refractory periods [10].

As a compromise, we consider a situation in which subjects are externally paced but the BCI is able to determine on which prompting cycles control commands are issued. A new BCI paradigm which has sensitive time slots to decode whether users want to initiate commands is investigated. Based on our semi-self-paced BCI system, users volitionally determine in which time slot to issue an actual command (e.g. left vs. right). Moreover, removing the ‘WHEN’ issue (time slot when an actual command should be given) and separating command initiation (subject’s choosing whether or not to issue an actual command) from actual commands will lower user control complexity and BCI computation. In asynchronous BCI systems based on steady-state visually evoked potentials (SSVEP), stimuli are constantly flicking in order to evoke potential control commands for external devices [18]. However, constant flickering can be quite uncomfortable for users. Moreover, even when users do not want to issue any commands, the flicking light still goes on which can evoke potentials to be detected as commands [19], thus leading to false positives. In such situations, the proposed semi-self-paced paradigm could be used to turn on and turn off flicking which will be comfortable for users and decrease the false positive rate. An asynchronous BCI control of a wheelchair in a virtual environment has been studied with a tetraplegic subject [20]. However, that BCI used only two command states, which limited the direction of movement. As more classes of motor imagery are included, complicated signal processing [21] or protocol of classification methods [22] are needed, requiring heavy computation during the periods when the subject is resting. Our paradigm largely mitigates the signal detection problem of fully asynchronous BCIs while relaxing the requirement that subjects issue control commands on every cycle. In order to implement our proposed BCI paradigm, we examined the following questions in this paper:

1. Is scalp EEG sufficient for distinguishing between an “initiation” state, when subjects are preparing to make a choice, and a “non-initiation” state during which subjects are engaged in otherwise identical sensory and motor task contingencies but not required to make a choice?
2. Can EEG spectral power features discriminate between initiation and non-initiation mental states based on single trials with an accuracy of at least 70%, a threshold which is acceptable in practical BCI applications [13]. We compared the classification accuracy achieved using power in the delta, theta, alpha and beta frequency bands [24].
3. Recordings over which brain areas will be the most effective in this semi-self-paced BCI application using EEG spectral power as the classification feature? We are particularly interested in whether EEG recordings over certain brain regions are superior in discriminating initiation and non-initiation states, or whether there is uniform discriminability across brain regions.

## Materials and Methods

### A. Semi-self-paced BCI Paradigm

**Figure 1:**
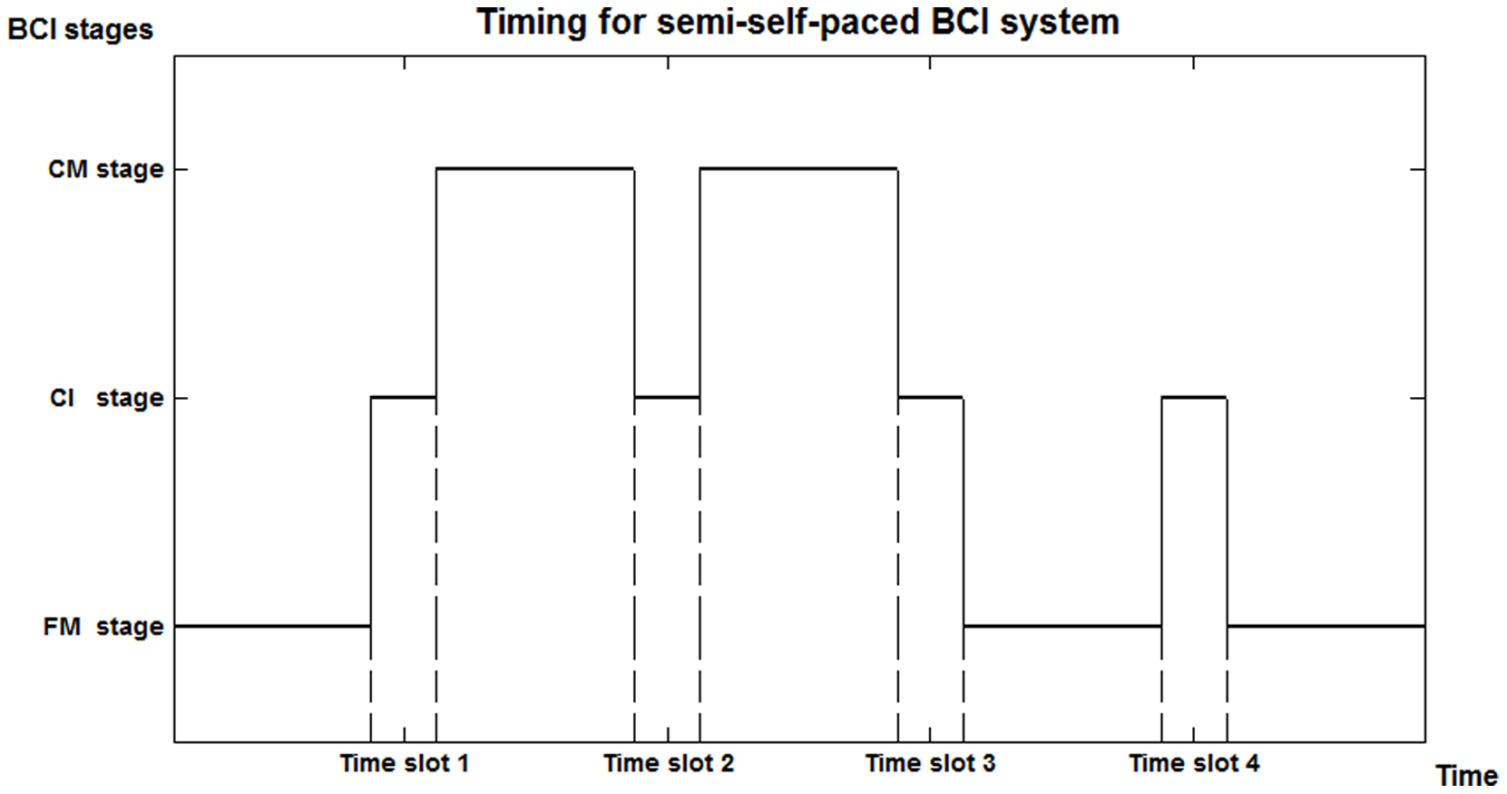
Timing for a semi-self-paced BCI system with three stages. For a semi-self-paced BCI system, there are three stage: free mental (FM), control initiation (CI), and command (CM) stages. At each pre-set time slot, i.e., control initiation stage, the user can decide to enter the command stage or free mental stage. The figure shows that the user determined to switch to the command stage from the previous free mental stage at time slot 1. At time slot 2, the user chose to stay at the previous stage, i.e. continuing to provide BCI commands. When time slot 3 arrived, the user switched to the free mental stage. The user remained in this stage at time slot 4, such that the user was free from the mental tasks required in controlling the BCI.

Semi-self-paced BCI is a very practical and user-friendly mode of operation comprising three stages: an unrestricted cognitive stage (“free mental stage”), a control initiation stage and a command stage. Users are not consciously controlling their brain activity in the free mental stage so that they can be free from the mental tasks required in controlling the BCI. During the control initiation stage at pre-set time slots, users have the option to determine whether they want to issue a command by entering the command stage, or they can stay in the free mental stage. Once users are in the command stage, they can affect the BCI output to give commands and communicate with the outside. Figure 1 depicts the timing for a semi-self-paced BCI system.

### B. Subjects

Nineteen normal undergraduate students recruited through the University of California at San Diego (UCSD) Department of Psychology participated in the study. After detailed explanation of the procedures, all subjects provided written informed consent consistent with the Declaration of Helsinki. The study was approved by the UCSD Institutional Review Board. All subjects declared no history of neurological disorder. All subjects were right handed according to Edinburgh handedness inventory [25] and had normal or corrected to normal vision. Data of seven subjects were removed because of technical problems with EEG acquisition for six subjects and one subject allegedly had prior experience with similar experiments. Five of the remaining twelve subjects (age range 19–23, mean 20.3, SD 1.2) were female.

### C. Task Design

Subjects were presented with a series of 512 trials in which they chose between abstract visual images with a possibility of accruing a small cash reward in each trial. The images presented in each trial were selected from among four possible images, each with a fixed probability of producing an identical reward value. In order to maximize their earnings, subjects had to learn through trial-and-error which images were more likely to pay off. The stimulus-reward contingencies and corresponding rewarded learning elements of the task are outside the scope of the present study. See Peterson et al. [26], [27] for full details on the experimental task design.

**Figure 2:**
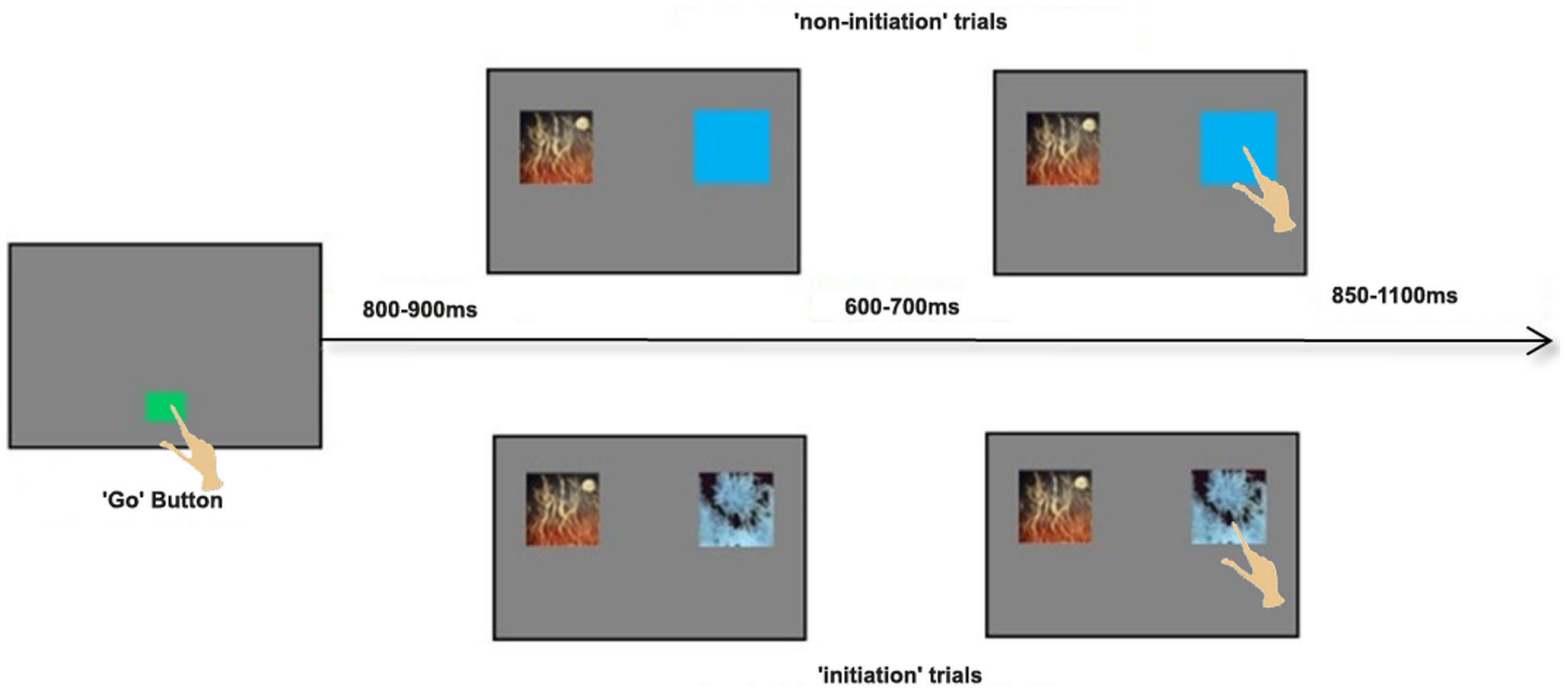
Timeline of each trial(modified based on Peterson et al., 2011 [26]). Subjects initiated each trial by pressing the green “go button” square in the lower middle of the touch monitor. After 800–900 ms, a square visual image appeared in each of the two upper corners of the touch monitor. On the non-initiation trials, subjects were presented with a solid blue square and one of four abstract images. They were instructed to always choose the abstract image. On the initiation trials, subjects were presented with two of the abstract images and were told to “choose the image that is more likely to pay off”. Subjects required 600–700 ms to identify an image before they moved to choose it. There was an 850–1100 ms delay before the start of the next trial.

Subjects were seated in front of a 19” touch monitor in sufficiently close proximity to allow comfortable reaches to both upper corners. The touch monitor was placed on a table with the top approximately 45° back from vertical. As depicted in Figure 2, subjects initiated each trial by pressing the green “go button” square in the lower middle of the touch monitor. After 800–900 ms, a 3 inch square visual image appeared in each of the two upper corners of the touch monitor. Subjects required 600–700 ms to identify an image before they moved to choose it. There was an 850–1100 ms delay before the start of the next trial. Actual durations of each time interval specified above were chosen randomly from a uniform distribution in each trial.

In this implementation, the task included two randomly interleaved trial types: “non-initiation” and “initiation” trials. On the non-initiation trials, subjects were given an “instructed choice”. They were presented with a solid blue square and one of four abstract images. They were instructed to always choose the abstract image. On the initiation trials, subjects faced a two-alternative forced choice. They were presented with two of the abstract images and were told to “choose the image that is more likely to pay off”. There were no initiation trials on which the two images were identical. We fully counterbalanced the number of presentations of each image and the side on which they were presented. Maximum run lengths were three initiation trials, five non-initiation trials, three non-initiation trials with the abstract image on the same side, and five trials containing the same image on either side. In this paper, the analysis is focused on the period between image onset and the start of movement to select the image. During this interval, subjects compared and identified images in their minds without any overt movements for both trial types.

### D. EEG & EOG Signal Recording and Preprocessing

Scalp EEG was measured throughout the experiment at 512 Hz using a 70-channel active electrode EEG system (Biosemi, Inc.), including 64 scalp electrodes, one electrode on each of left and right mastoid, electrodes above and below the right eye for vertical electrooculogram (VEOG), and lateral to the outer canthus of each eye for horizontal electrooculogram (HEOG).

EEG signals were segmented into 800 ms epochs, i.e. [−200 600] ms time-locked to the onset of the two images for each initiation and non-initiation state. No movement occurred in [−200 600]ms based on the analysis that subjects required at least 600 ms to identify a selection before they moved to choose it. Subsequently, EEG epochs with excessive amplitude (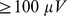) were rejected from further analysis. Epoched data were then passed to a third-order Butterworth bandpass filter in the frequency range of 0.5–30 Hz. Each trial contained signals from images onset to 600 ms, i.e. [0 600] ms. Signals during this interval were baseline-normalized by subtracting the mean EEG voltage in [−200 0] ms. For each trial, a common average reference [28] signal, which was computed as the average voltage amplitude of all 64 EEG signal channels, was subtracted from the signal from each EEG electrode in order to remove the unnecessary noise in the EEG data acquired by the active electrodes [29].

Independent components analysis (ICA) decomposition using extended Infomax [30] was applied to the EEG data. We used DIPFIT [31] with the canonical Montreal Neurological Institute boundary element head model to fit each independent component (IC) to generate a single equivalent current dipole. In order to remove eye and muscle movement artifacts, ICs whose dipoles localized outside the brain volume or had residual variance larger than 20% were rejected. Then the remaining ICs were projected back to the original electrode space to extract classification features without further rejection.

EOG signals were also first segmented into [−200 600] ms epochs relative to the onset of the two images and then filtered by the same third-order Butterworth bandpass filter in the frequency range of 0.5–30 Hz. The average voltage in [−200 0] ms was subtracted from the EOG data in [0 600] ms, as well, to generate the EOG trials.

### E. Feature Extraction

Seven EEG electrode clusters spanning the majority of the scalp as well as one EOG cluster were selected for decoding analyses: medial frontal to parietal brain areas (AFz, Fz, FCz, Cz, Pz, CPz), frontal (F1, F3, F5, F2, F4, F6), right-parietal (CP4, CP6, P4, P6, P8, TP8), left-parietal(CP3, CP5, P3, P5, P7, TP7), left-motor (FC1, FC3, FC5, C1, C3, C5), right-motor (FC2, FC4, FC6, C2, C4, C6), parietal-occipital (PO7, PO3, O1, O2, PO4, PO8), and the EOG cluster (four electrodes: two above and below the right eye, and two lateral to the outer canthus of each eye). For each [0 600] ms trial, the band power was calculated with fast Fourier transform (FFT) at each selected electrode in frequency bands ranging from 1.67 Hz to 30 Hz with bandwidth of 1.67 Hz. Each 1.67 Hz band power in the delta (1.67–4 Hz), theta (5.01–8.35 Hz) and alpha (9–13.36 Hz) frequency bands were directly used as discriminant features, resulting in 2, 3 and 3 features respectively for each electrode. As the bandwidth is much larger in beta (14–30 Hz), the beta band powers were averaged in each pair (15.03,16.7) Hz, (18.37,20.04) Hz, (21.71,23.38) Hz, (25.05,26.72) Hz and (28.39,30.06) Hz to derive 5 features for each electrode. There are six electrodes in each EEG cluster and four electrodes in the EOG cluster. The resulting feature dimensionalities in each EEG cluster, taking into account the number of features per band × the number of electrodes, were and 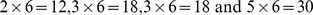 respectively. Similarly, the feature dimensionalities in the EOG cluster were 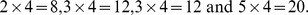 The electrode cluster that contained the greatest amount of information differentiating initiation and non-initiation states would be the one that achieved the highest classification accuracy.

### F. Classifiers and Statistical Comparisons

Support vector machine (SVM) with Gaussian radial basis function (RBF) kernel implemented with the LIBSVM [32] Matlab toolbox was chosen based on previous comparisons of classifiers for scalp EEG-based BCIs [33]. For each subject, randomly selected sets of 80% of the initiation trials and 80% of the non-initiation trials were assigned to the training set, and the remaining 20% to the testing set. In the training stage, the grid-search algorithm was performed to identify optimal SVM soft margin parameter 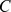 and the width of Gaussian kernel 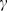 using 10-fold cross validation. The parameters 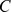 and 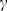 were searched in the range 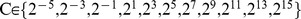 and 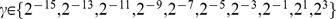. The discriminant model trained based on all trials in the training set using Gaussian RBF SVM with optimal parameters was tested in the testing set. Prediction accuracy was measured by the percentage of correctly classified trials of the testing set. A two-way ANOVA was performed on the classification accuracy with EEG clusters (seven brain regions and one EOG cluster), and frequency bands (delta, theta, alpha, beta) as factors. We used a significance threshold of 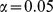 for all statistical tests. The Shapiro-Wilk test was used to prove that the classification accuracy fulfills the Gaussian distribution.

## Results

### A. Band Power Differences between Initiation and Non-initiation States

**Figure 3:**
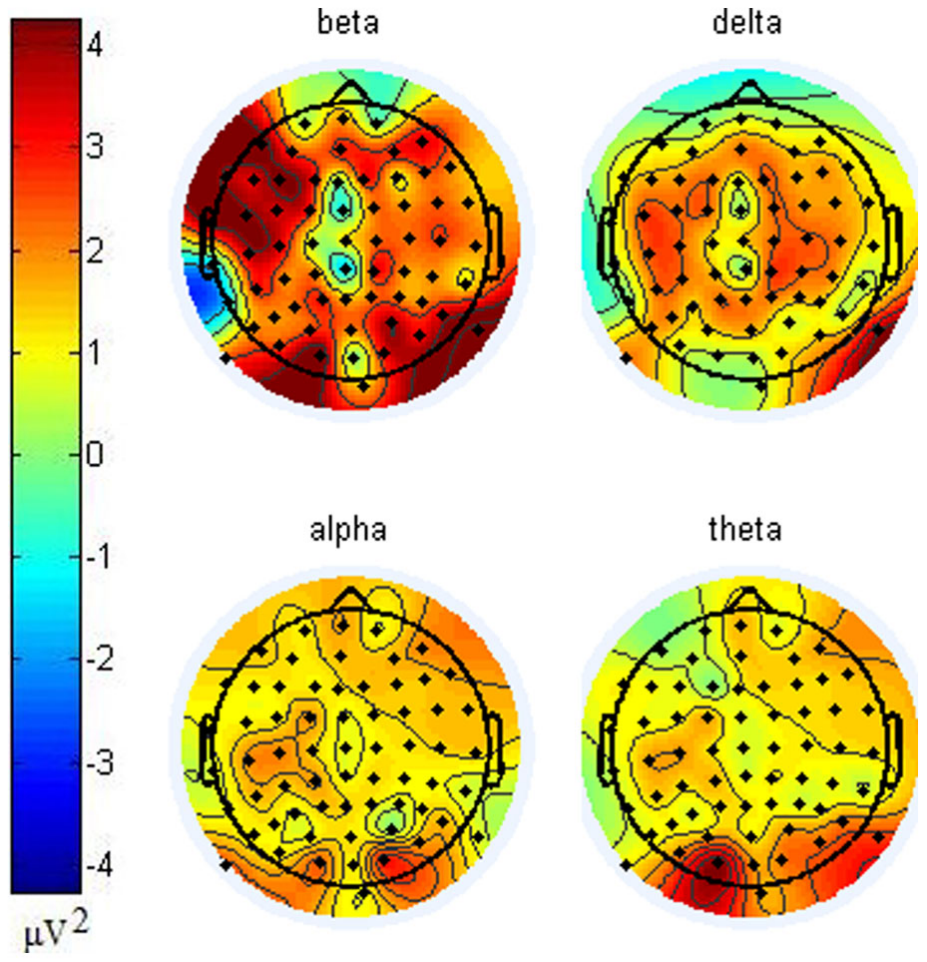
Topologies of band power differences between initiation and non-initiation states. Power differences were calculated by subtracting powers in non-initiation state from initiation state in each frequency band in each electrode. Beta band powers from various clusters were observed to be higher in initiation state than in non-initiation state. While larger delta band power differences occurred in the back of frontal, motor and front of parietal clusters. Band powers in occipital lobe for alpha and theta bands were higher in initiation state.

Figure 3 presents topologies of power differences that were calculated by subtracting power in non-initiation state from initiation state in each frequency band. Beta band powers from various clusters were observed to be higher in the initiation state than in the non-initiation state. The largest differences in beta band power between these two states occurred in occipital and left frontal/motor clusters, which was consistent with [34]. While larger delta band power differences occurred in the posterior portions of frontal and motor clusters, and the anterior portion of parietal clusters. Band power differences in alpha and theta bands were larger in the occipital lobe. The heavier working load in initiation state would induce higher alpha and theta band power [35], [36].

### B. Prediction Accuracy Comparison over Frequency Bands and Electrode Clusters

**Figure 4:**
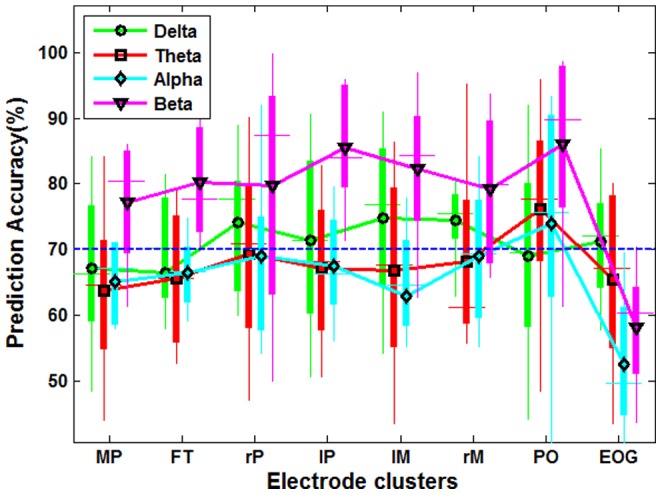
Prediction accuracy averaged across all subjects using band power in the delta, theta, alpha and beta frequency bands. The lower and upper edges of each box are the 25th and 75th percentiles of prediction accuracy, while lower and upper whiskers represent the worst and best accuracy. The black symbol in each box is the average prediction accuracy across all subjects. MR, FT, rP, lP, lM, rM, PO and EOG represent medial regions, frontal, right-parietal, left-parietal, left-motor, right-motor, parietal-occipital and the EOG cluster respectively.

Figure 4 presents the results from comparing the classification accuracies achieved with the features based on four standard frequency bands over seven brain areas and one EOG electrode cluster using all data in the time interval 0–600 ms. The results reported here were based on the statistics averaged over all twelve subjects. A threshold of 70% accuracy deemed to be acceptable in practical BCI application [23] was adopted in this paper as well. Band power in the beta frequency band provided the best performance in each brain region, with an accuracy ranging from 78%–86% in classifying initiation and non-initiation mental states. Delta band power over right-parietal, left-parietal, left-motor, right-motor and EOG clusters also achieved classification accuracies higher than the threshold. Moreover, band power in theta and alpha band recorded over parietal-occipital area yielded acceptable performance for practical BCI applications.

Table 1 presents the results of the two-factor ANOVA statistical analyses comparing the eight electrode clusters and four standard frequency bands used in Figure 4. Factors of electrode cluster and frequency band had significant effects on prediction accuracy. Table 2 presents the post hoc analyses of the pair-wise comparisons of eight different electrode clusters, and Table 3 presents the pair-wise comparisons for the four frequency bands. Post hoc tests indicated that prediction accuracies achieved over parietal-occipital, left & right parietal and left & right motor areas were superior to those over medial brain regions and EOG signals. Table 3 shows that beta band power performs better than other frequency bands in distinguishing initiation state from non-initiation state.

**Table 1:** Statistical Anova results for eight electrode clusters and four standard frequency bands.

Factor(s)	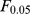	F	P
Electrode Clusters	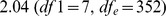	3.7	0.0008
Standard Frequency Bands	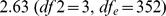	12.33	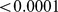
Electrode Clusters x Standard Frequency Bands	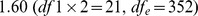	1.09	0.3594

**Table 2:** Post hoc analysis of Anova results for eight electrode clusters.

	PO	rP	lP	rM	lM	FT	MR	EOG
PO						*	*	*
rP							*	*
lP							*	*
rM							*	*
lM							*	*
FT								
MR								
EOG								

Prediction accuracy of electrode clusters are in descending order from left to right and from top to bottom. Tukey’s range test was used for all post hoc analysis. Conditions that are significantly different are marked with “*”. PO, rP, lP, rM, lM, FT, MR and EOG represent parietal-occipital, right-parietal, left-parietal, right-motor, left-motor, frontal, medial regions, and the EOG cluster respectively.

**Table 3:** Post hoc analysis of Anova results for four standard frequency bands.

Standard Frequency Bands	Beta	Delta	Theta	Alpha
Beta		*	*	*
Delta				
Theta				
Alpha				

Prediction accuracy in four standard frequency bands are given in descending order from left to right and top to bottom. Tukey’s range test was used for all post hoc analysis. Conditions that are significantly different are marked with “*”.

### C. Prediction Accuracy Comparison over Subjects

**Figure 5:**
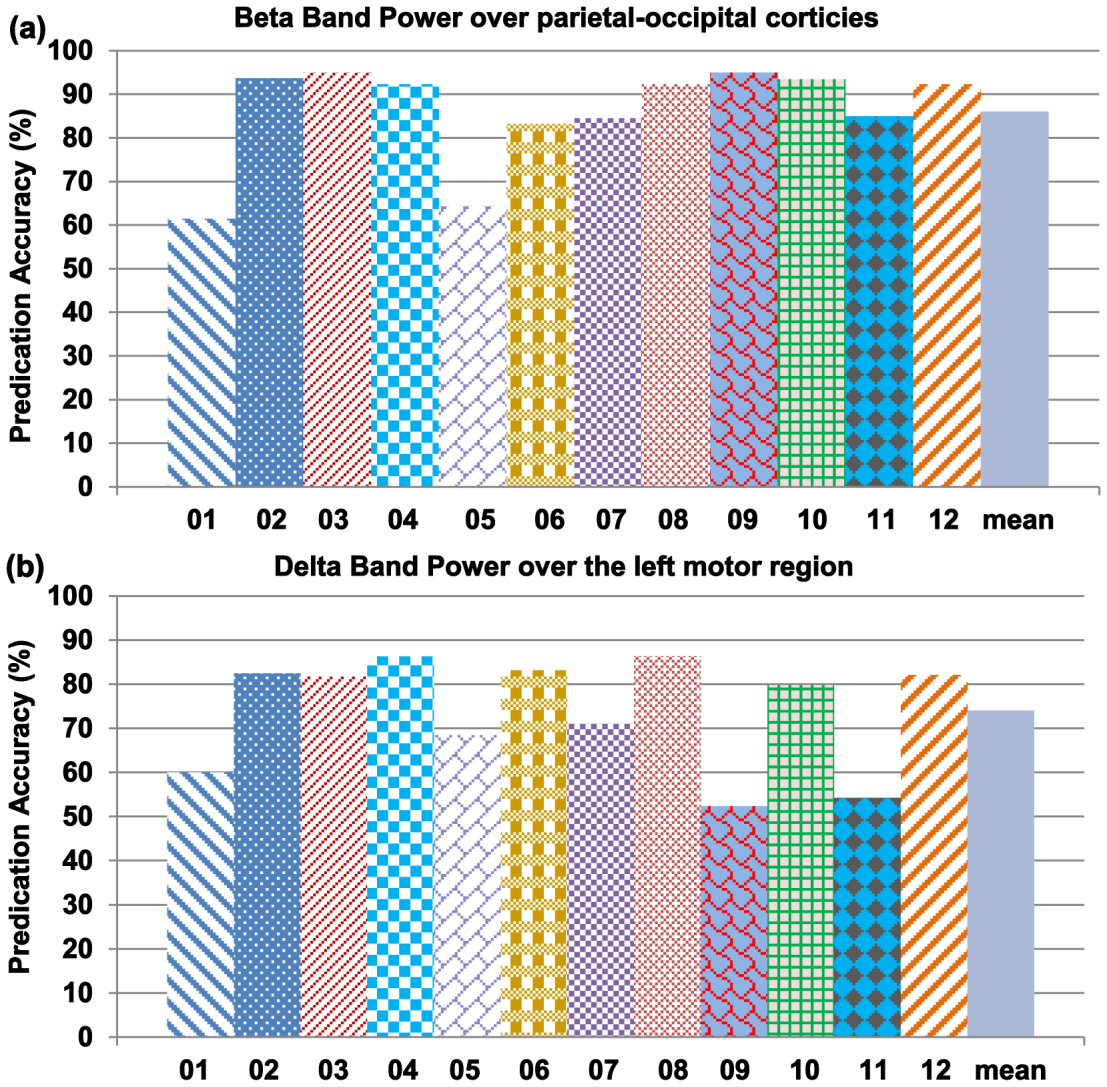
Prediction accuracy achieved for each subject using beta band power. Prediction accuracy achieved for each subject using beta band power over the parietal-occipital area (a) and delta band power over the left motor area (b). Beta band power over the parietal-occipital area and delta band power over the left motor area both achieved an average accuracy greater than 70%, with some subjects achieving nearly 100% accuracy using beta band power recorded over parietal-occipital corticies.

Individual differences were present when the same classification feature was used across subjects. Figure 5(a) shows the achieved prediction accuracy for each subject using beta band power over the parietal-occipital region, which obtained the highest accuracy (86%) averaged over all subjects (Figure 4). Ten out of twelve subjects yielded an accuracy of higher than 80%, six of which even achieved higher than 90% accuracy. However, two subjects only obtained 60–65% accuracy. Figure 5(b) illustrates the accuracies predicted by delta band powers in the left motor area which achieved the highest accuracy (Figure 4) averaged among all EEG clusters in the delta frequency band. Subject 4 achieved the best performance with 85% accuracy, while subjects 9 and 11 yielded only 55% accuracy, which was near chance level.

## Discussion

In this study, we investigated the fundamental problem of separating initiation states from non-initiation states, demonstrating the viability of a new approach for BCI design in which subjects are externally paced but allowed to determine on which prompting cycles control commands are issued. Since the proposed paradigm is novel, we evaluated it off-line prior to any on-line application. Using SVM classification and EEG spectral features, we could distinguish between the initiation state, when subjects were preparing to make choices, and non-initiation states during which subjects were engaged in otherwise similar task contingencies but not required to make a choice. Higher band power was investigated in the initiation state, especially in beta and delta bands. Beta band power yielded the highest prediction accuracy among four standard frequency bands. Performance was above 70% correctly classified trials averaged over all subjects for all EEG clusters, with the highest accuracy of 86% achieved at the parietal-occipital area. Moreover, delta band power over parietal and motor areas also achieved higher than 70% accuracy, and thus could be used as an alternative feature to discriminate initiation and non-initiation states. Individual differences were present using the same classification feature across subjects. Two subjects with the lowest classification accuracy using delta band power over the left motor regions achieved much higher prediction accuracy using beta band power over parietal-occipital cortices. To achieve high classification accuracy, subject-specific weighting for different frequency bands in different brain regions would help in future BCIs customized to individuals. Although spectral power of EEG provided good classification in our study, additional future studies are required to validate the generality of this finding across tasks, as well as across other types of non-invasive and invasive BCIs.

**Figure 6:**
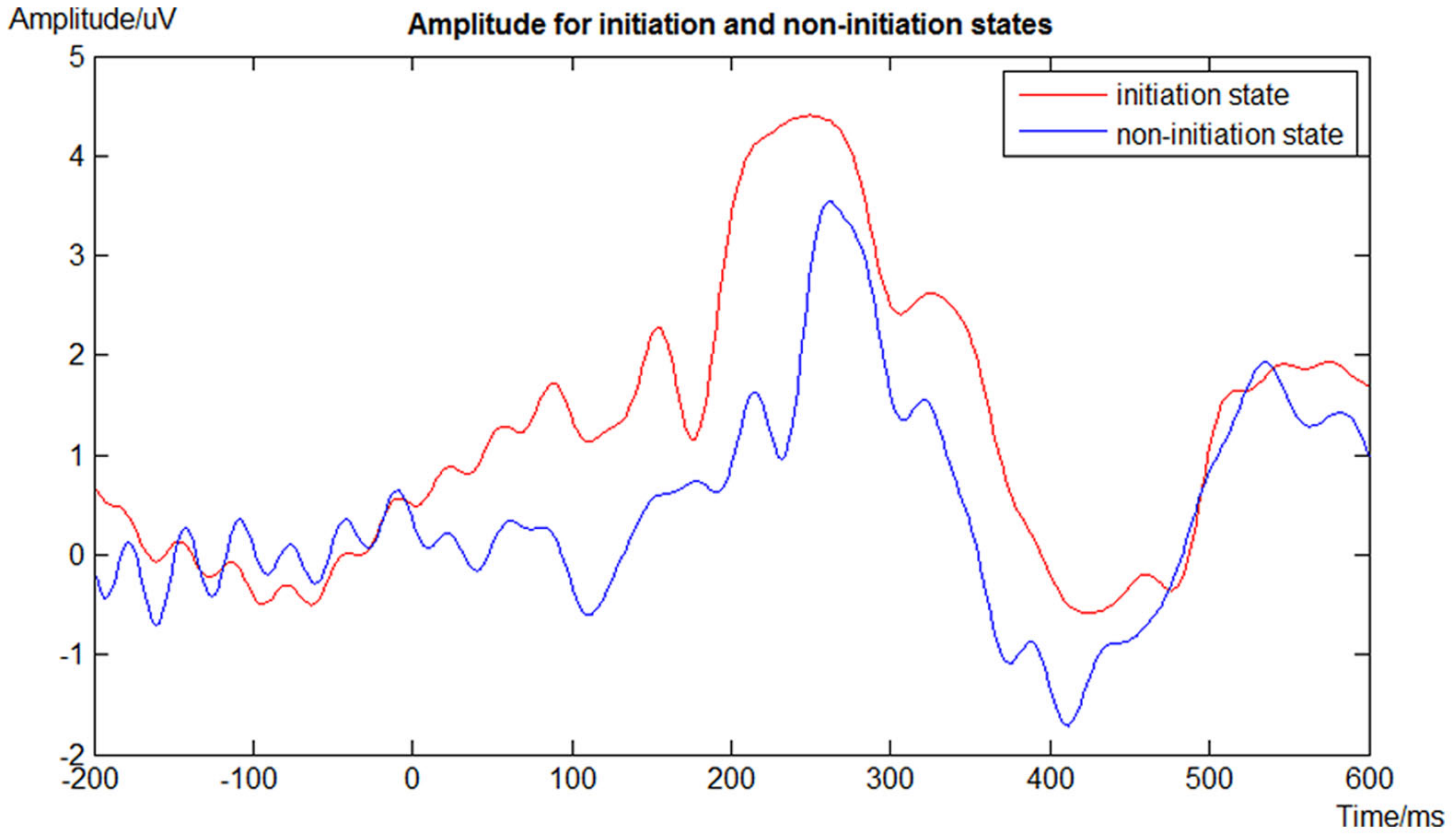
Time domain amplitude in [−200 600]ms respect to stimulus onset for initiation and non-initiation states. A sample EEG waveform average across trials from left parietal cluster (CP3, CP5, P3, P5, P7, TP7) for one representative subject was shown, in which P300 potential could be observed in both states. The appearance of two natural images generates higher P300 response compared to the condition with one blue square. Blue line represents the averaged amplitude in non-initiation state; red line represents the averaged amplitude in initiation state.

One notable difference between the two trial types in our task is that, in the non-initiation trials, one of the two visual stimuli presented to subjects is an instructional visual cue indicating that it is a non-initiation task. The cue is a visual stimulus (solid blue square) that is visually distinct from the set of stimuli from which subjects choose in the initiation trials. The dimensions, location, and onset/offset timing characteristics of the cue are otherwise identical. The paradigm requires subjects analyzing the images appearing on the monitor screen and making the proper selection. The process may involve various brain structures and modulations besides EEG power spectra. The time-domain amplitudes for “initiation” and “non-initiation” states are presented in Figure 6. Since the subjects have to react to the more valuable image in the initiation state, the appearance of such stimuli generates a higher P300 potential compared to the non-initiation state. Considering the length of the feature window (600 ms), the differences of the P300 amplitude in the “initiation” and “non-initiation” states might be one of origins for the differences in power spectra between these two experimental conditions. There might be other evoked potentials differences as well, which could be investigated in the future.

Based on our results, an alternative way to implement an online semi-self-paced BCI system can be described as follows. Similar to our experiment, an alerting green ‘go’ square can be presented 800 ms ahead of control initiation stage. Assume the BCI starts at the free mental stage. Two abstract images and a solid blue square appear in random order in the screen in each pre-set time slot. Users compare two abstract images carefully to identify the more valuable one, if they want to switch to the command stage. Otherwise, users simply think about choosing the solid square to stay in the free mental stage. Moreover, control initiation time slots could be presented between intervals of two actual commands during the command stage, so that users can switch to the free mental stage conveniently. Patients don’t need to learn and remember extra control information when using similar visual patterns during all the BCI stages, which can decrease the burden of memory. Only mental responses are required to control the whole proposed BCI stages.Meanwhile the implementation of the proposed on-line BCI system is, in our opinion, not more difficult than for other BCIs.
